# Supplementary material for: Cyclic, Condition-Independent Activity in Primary Motor Cortex Predicts Corrective Movement Behavior
Source: eNeuro. 2022 Apr 13;9(2):ENEURO.0354-21.2022. doi: 10.1523/ENEURO.0354-21.2022 (PMC9014981; doi:10.1523/ENEURO.0354-21.2022)
Supplement: Extended Data 1 — MATLAB code to calculate the CIφ is available on GitHub. Since the trial data contains corrective movements in addition to the large initial movements that were not precisely time aligned to trial events for averaging CI neural activity, we developed a novel algorithm to iteratively average the firing rates, calculate CIφ, then average the firing rates again based on the CIφ. This iterative process involves three steps: (1) each unit’s firing rate is averaged across all trials to determine its CI firing rate; (2) dimensionality reduction is performed using PCA and jPCA on the CI firing rates to identify the neural plane with the most rotational/cyclic CI activity; (3) the instantaneous phase is calculated using the Hilbert transform on the first two jPC dimensions for all data points. MATLAB code is available on GitHub. Further details are available in the Readme document attached to the code. Download Extended Data 1, ZIP file. [file enu-eN-NWR-0354-21-s02.zip › ExtendedData1/Readme.pdf]

Calculation of jPCA plane and Condition-Independent Phase

Since the trial data contains corrective movements in addition to the large initial movements that were not precisely time aligned to trial events for averaging condition-independent neural activity, we developed a novel algorithm to iteratively average the firing rates, calculate CIφ, then average the firing rates again based on the CIφ. This iterative process involves three steps: i) Each unit's firing rate is averaged across all trials to determine its condition-independent firing rate. ii) Dimensionality reduction is performed using PCA and jPCA on the condition-independent firing rates to identify the neural plane with the most rotational/cyclic condition-independent activity. iii) The instantaneous phase is calculated using the Hilbert transform on the first two jPC dimensions for all data points. A schematic of the iterative algorithm is shown below.

i) Trial averaging to identify condition-independent activity

The condition-independent neural activity is the average firing rate for each recorded spiking unit for all experimental trials regardless of the movement condition (ie target location). For classic neurophysiology experiments, this can be calculated by averaging time-aligned data. However, since trials were of varying durations and many included corrective movements, simple time alignment of trials was difficult. Data was therefore aligned based on each time-point's calculated CIφ rather than absolute time.

Since CIφ depends on the averaged condition-independent activity and the condition-independent activity was averaged by CIφ alignment, an iterative approach was required. An initial estimate using simple time-alignment averaging of all initial and corrective submovements was used for the first iteration. For each subsequent iteration, the condition-independent firing rates for each unit were calculated by averaging all data points when the CIφ values were similar. The average firing rate was estimated using a sliding window of CIφ values with a step size of $\pi/50$ and a window size of $\pi/25$ to generate 100 equally spaced samples ranging from $-\pi$ to π.

ii) PCA and jPCA to identify rotational/cyclic neural activity

Next, the condition-independent firing rates were submitted to PCA and jPCA (Churchland et al., 2012) to identify the neural dimensions with the most cyclic activity. PCA was performed on the high-dimensional neural space to reduce the condition-independent firing rates to the six dimensions with the most variance. jPCA was then performed on this six-dimensional space. jPCA is a dimensionality reduction technique to identify the neural planes with the most rotational activity and is more fully described in Churchland et al (2012). Briefly, jPCA fits a first order dynamical system model to the neural activity :

$$\dot{X} = AX$$

to predict change in firing rate ($\dot{X}$) based on the current firing rate (X). The transform matrix of this model (A) can be separated into a symmetric matrix representing pure scaling and a skew-symmetric matrix representing pure rotational dynamics. By taking the eigendecomposition of the skew-symmetric matrix, we obtain pairs of purely imaginary eigenvalues and corresponding eigenvectors that define planes of rotation in the neural space rank-ordered from greatest to least rotation. In the present analysis, only the first plane with the greatest condition-independent rotation was used and we defined the two dimensions of the plane as CIx and CIy. Additionally, to obtain a consistent CIφ across recording sessions, CIx was defined as the dimension in the CIx/CIy plane with the most variance. The positive CIx direction was defined as having more positive than negative coefficients in the neural space which corresponds to the direction where more units have an increased firing rate. Choosing this convention causes an increase in CIx to generally align with the onset of initial movement, since a majority of units increase firing rates at the onset of movement.

iii) Instantaneous phase estimate

Finally, the instantaneous phase were estimated by i) bidirectional bandpass filtering of the activity in both the CIx and CIy dimensions between 0.5-5 Hz with a 1st order Butterworth filter, ii) performing the Hilbert transform of both filtered signal (s_x and s_y) to generate a transformed signal

($\hat{s}_x$ and $\hat{s}_y$) that is a 90˚ phase shift of every Fourier component in the frequency domain to create an analytic representation of CIx and CIy, and iii) calculating the angle of the resulting analytic signal ($s_1 + i\hat{s}_1$) to estimate the instantaneous phase in each dimension, and iv) which we then sum (with a $\frac{\pi}{2}$ phase shift added to ϕy) to obtain a single instantaneous phase estimate for the neural activity within the plane which we call the condition-independent phase ($CI\varphi$).

i) $s_x(t) = filt_{0.5-5Hz}[CIx(t)];$

ii) $\hat{s}_x(t) = \mathcal{H}[s_x(t)]$

iii) $\phi x\ (t) \ = \ \arg\{s_x(t) + i\hat{s}_x(t)\}$

iv) $CI\phi\ (t) = \ \phi x + (\phi y + \frac{\pi}{2})$

$Note -$ i, ii, and iii are performed for both x and y

The bandpass filtering reduces the low-frequency drift and high-frequency variability in CIx to generate a more consistent subsequent phase estimate. The condition-independent phase (CIφ) thus represents the instantaneous phase in the dimension of the neural space that has the most cyclic, condition-independent activity.

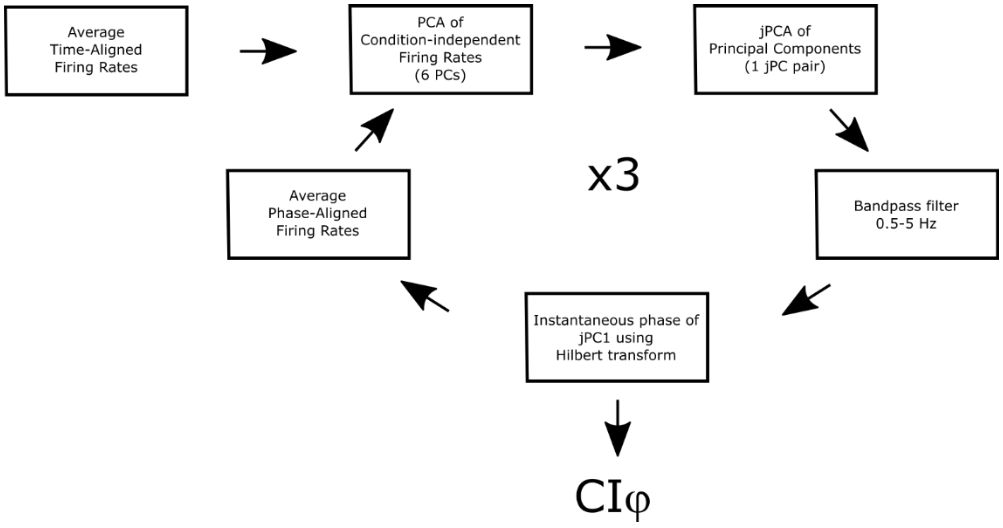

Schematic of CIϕ estimation. The cyclic condition-independent neural activity was estimated using an iterative technique. After initial time-alignment and averaging of the firing rates, PCA and jPCA was performed to estimate the dimensions neural activity with the greatest cyclic condition-activity were identified. Using the phase of this first jPC dimension, the firing rates were realigned for averaging. The PCA and jPCA were again performed a total of three times. The final CIϕ was then used for the presented analysis.
